# IMPACT OF THE COVID-19 PANDEMIC ON THE EMERGENCY SURGICAL TREATMENT OF COLORECTAL CANCER

**DOI:** 10.1590/0102-672020230075e1793

**Published:** 2024-02-05

**Authors:** Danilo Toshio KANNO, Roberta Laís Mendonça de MATTOS, Rayama Moreira SIQUEIRA, José Aires PEREIRA, Fábio Guilherme CAMPOS, Carlos Augusto Real MARTINEZ

**Affiliations:** 1 Universidade São Francisco, Postgraduate Program in Health Sciences – Bragança Paulista (SP), Brazil;; 2 Universidade Estadual de Campinas, Department of Surgery, Postgraduate Program in Surgical Sciences – Campinas (SP), Brazil;; 3 Universidade de São Paulo, Department of Gastroenterology – São Paulo (SP), Brazil;; 4 Universidade Estadual de Campinas, Department of Surgery – Campinas (SP), Brazil

**Keywords:** Colorectal Neoplasms, COVID-19, Emergencies, General Surgery, Intestinal Perforation, Intestinal Obstruction, Neoplasias Colorretais, COVID-19, Emergências, Cirurgia Geral, Perfuração Intestinal, Obstrução Intestinal

## Abstract

**BACKGROUND:**

Colorectal cancer (CRC) is the most common malignancy of the gastrointestinal tract and the third most common type of cancer worldwide. The COVID-19 pandemic, during the years 2020 and 2022, increased the difficulties in offering adequate early diagnosis and treatment to CRC patients worldwide. During this period, it was only possible to treat patients who evolved with complications, mainly intestinal obstruction and perforation.

**AIMS:**

To assess the impact of the COVID-19 pandemic on the treatment of patients with CRC.

**METHODS:**

A review of data from a total of 112 patients undergoing emergency surgical treatment due to complications of CRC was carried out. Of these, 78 patients underwent emergency surgery during the COVID-19 pandemic (2020/2021), and 34 were treated before the pandemic (2018/2019). Ethnic aspects, clinical symptoms, laboratory tests, histopathological variables, intra and postoperative complications, and 90-day postoperative follow-up were compared between the two groups.

**RESULTS:**

Between the years 2018 and 2019, 79.4% (27/34) of patients had intestinal obstruction, while 20.6% (7/34) had intestinal perforation. During the period of the COVID-19 pandemic (2020/2021), 1.3% (1/78) of patients underwent surgery due to gastrointestinal bleeding, 6.4% (5/78) due to intestinal perforation, and 92.3% (72/78) due to intestinal obstruction. No statistically significant differences were recorded between the two groups in ethnic aspects, laboratory tests, type of complications, number of lymph nodes resected, compromised lymph nodes, TNM staging, pre or intraoperative complications, length of stay, readmission, or mortality rate. When considering postoperative tumor staging, among patients operated on in 2018/2019, 44.1% were classified as stage III and 38.2% as stage IV, while during the pandemic period, 28.2% presented stage III and 51.3% stage IV, also without a statistically significant difference between the two periods. Patients operated on during the pandemic had higher rates of vascular, lymphatic and perineural invasion.

**CONCLUSIONS:**

The COVID-19 pandemic increased the rate of complications related to CRC when comparing patients treated before and during the pandemic. Furthermore, it had a negative impact on histopathological variables, causing worse oncological prognoses in patients undergoing emergency surgery.

## RESUMO

**RACIONAL:**

O câncer colorretal (CCR) é a doença maligna mais comum do trato gastrointestinal sendo o terceiro tipo de câncer mais comum em todo o mundo. A pandemia de COVID-19 durante os anos de 2020 e 2022 aumentou as dificuldades em se oferecer diagnóstico e tratamento precoce adequado aos pacientes com CCR em todo o mundo. Nesse período, só foi possível tratar os pacientes que evoluíram com complicações representadas, principalmente, pela obstrução e perfuração intestinal.

**OBJETIVOS:**

Avaliar o impacto da pandemia de COVID-19 no tratamento de pacientes com CCR.

**MÉTODOS:**

Foi realizada uma revisão dos dados de um total de 112 pacientes submetidos ao tratamento cirúrgico de urgência devido complicações do CCR. Destes, 78 pacientes foram submetidos a cirurgia de emergência durante o período da pandemia de COVID-19 (2020/2021), e 34 pacientes foram operados no período anterior à pandemia (2018/2019). Aspectos étnicos, sintomas clínicos, exames laboratoriais, variáveis histopatológicas, complicações intra e pós-operatórias e acompanhamento pós-operatório de 90 dias foram analisados comparando os dois grupos.

**RESULTADOS:**

Entre os anos 2018 e 2019, 79,4% (27/34) dos pacientes apresentaram obstrução intestinal enquanto 20,6% (7/34) perfuração intestinal. Durante o período da pandemia de COVID-19 (2020/2021) 1,3% (1/78) dos pacientes foram operados por hemorragia digestiva baixa, 6,4% (5/78) por perfuração intestinal e 92,3% (72/78) por obstrução intestinal. Não foram registradas diferenças estatisticamente significativas entre os dois grupos nos aspectos étnicos, exames laboratoriais, tipo de complicações, número de linfonodos ressecados, linfonodos comprometidos, estadiamento TNM, complicações pré ou intraoperatórias, tempo de internação, readmissão e taxa de mortalidade. Ao considerar o estadiamento tumoral pós-operatório, entre os pacientes operados em 2018/19, 44,1% foram classificados como estágio III e 38,2% como estágio IV, enquanto no período pandêmico, 28,2% apresentaram estágio III e 51,3% estágio IV, também sem diferença estatisticamente significativa entre os dois períodos. Doentes operados durante a pandemia apresentaram maiores índices de invasão vascular, linfática e perineural.

**CONCLUSÕES:**

A pandemia de COVID-19 aumentou as taxas de complicações relacionadas ao CCR, comparando pacientes tratados antes e durante a pandemia. Além disso, teve impacto negativo nas variáveis histopatológicas, causando piores prognósticos oncológicos em pacientes submetidos a cirurgias de emergência.

## INTRODUCTION

Colorectal cancer (CRC) is a well-known and common oncological disease, corresponding today to one of the most frequent malignant tumors worldwide^25^. In 2018, approximately 1.8 million new cases were diagnosed in the world, and approximately 881,000 deaths occurred, accounting for almost 10% of the total number of deaths when considering all malignant tumors^7,13^. Currently, epidemiological data show that CRC ranks third among malignant tumors and is the second most common in developed countries^6,19^. Every three years, the American Cancer Society provides an update of CRC statistics according to the incidence of population-based cancer registries and mortality from the United States National Center for Health Statistics. By 2023, approximately 153,020 individuals will be diagnosed with CRC, and 52,550 will die from the disease, including 19,550 cases and 3,750 deaths of individuals younger than 50 years^40^. For Brazil, it was estimated that for each year of the 2020/2022 triennium, there were 20,520 cases of colon and rectal cancer in men and 20,470 in women. These values correspond to an estimated risk of 19.63 new cases for every 100 thousand men and 19.03 for every 100 thousand women^19^.

Individuals in whom CRC is diagnosed in the early stages have higher rates of overall survival and disease-free survival, as well as a lower chance of recurrence and complications related to neoplasia^25^. Unfortunately, CRC presents as an emergency in a wide range of patients (from 7 to 40% of the total), but the vast majority of reports present a rate of approximately 30%^33^. Large bowel obstruction is the most common complication and represents almost 80% of the emergencies related to CRC, while perforation accounts for the remaining 20%^8,40^. Lower digestive bleeding is rare, responding to less than 1% of cases of CRC complications^33^.

The lack of early diagnosis, the difficulty of patient access to the reference centers for the treatment of the disease, and the difficulty of performing a colonoscopy represent the main factors responsible for the delayed diagnosis, the progression of the disease, and the emergence of complications that occur in tumors diagnosed in the most advanced stages^31^.

COVID-19 infection supposedly appeared in 2019 in Wuhan city, China^47^. It is believed that the infection may have been transmitted from wild animals to people^21^. From November/December 2019 onwards, the disease gained greater worldwide repercussions when it reached the European continent, particularly Italy, when the possibility of the virus transmission through the inroads was confirmed^23^. Due to the high number of people infected worldwide who developed severe forms of the disease, the COVID-19 pandemic caused the collapse of several health systems around the world^23^. It did not take long for the COVID-19 pandemic to reach South America, also causing an enormous impact on health systems^24^. In Brazil, the first case of COVID-19 was identified in the State of São Paulo in February 2020^14,30^. Despite having had a long time to develop coping and contingency strategies for the pandemic that was approaching, Brazil took ineffective preventive measures^43^. The lack of adequate management in the fight against the pandemic, combined with the chronic scrapping of health equipment in recent decades, had a great impact. Brazil quickly became one of the countries with the highest number of daily cases and one of the record holders of deaths^11^.

CRC management was severely impacted by the pandemic^15^. The screening strategies for this disease were seriously reduced during the pandemic period, with many patients remaining undiagnosed^11^. Most of the main essential services for diagnosis, for example, centers qualified to perform colonoscopies, during the critical period of the COVID-19 pandemic had their procedures suspended or substantially reduced^34^. As a consequence, the delay of diagnosis due to the impossibility of performing a colonoscopy also determined a significant increase in complications arising from CRC progression. Besides, the initial recommendations were to postpone elective surgical interventions due to the risk of contracting COVID-19 during hospitalization. This led to an increase in the number of patients presenting to the emergency room with complications such as lower digestive hemorrhage, colonic perforation, and large bowel obstruction, and much more severe symptomatology, leading to an increased risk of mortality and morbidity^34^. With the advance of the pandemic, an increase of 6% in the number of deaths related to CRC progression or its complications was shown^42^.

In Brazil, few studies have evaluated to date the impact that the COVID-19 pandemic had on the treatment of patients with CRC^1,12,14^. Besides, to our knowledge, none of these studies assessed the impact of the COVID-19 pandemic on patients who developed obstructive or perforative complications resulting from the progression of CRC.

Therefore, the objective of the present study is to evaluate the impact of COVID-19 by comparing patients treated before the pandemic (2018/2019) with those treated during the pandemic (2020/2021).

## METHODS

This study was authorized by the Ethical Committee in Research of Universidade São Francisco (process number: 4327982.6.0000.5514). All data acquired from patients’ medical records were analyzed after signed informed consent.

This retrospective cohort study was performed using routinely collected data from consecutive patients with a confirmed histopathological diagnosis of adenocarcinoma of the colon or rectum exclusively undergoing emergency surgery at São Francisco University Medical Hospital (HUSF), a tertiary referral teaching hospital located in the interior of the state of São Paulo, Brazil. Patients were grouped according to two distinct periods: the pre-COVID-19 pandemic (March 1, 2018 to October 1, 2019) and during the COVID-19 pandemic (March 1, 2020 to October 1, 2021).

The main analyzed variables included patient demographics (age, sex, ethnicity), clinical symptoms (pain, rectal bleeding, loss of weight, tenesmus), laboratory test results (hemoglobin, hematocrit, albumin, C-reactive protein), surgical technique aspects (presence of stoma, intraoperative and postoperative complications), surgeon’s specialty (general surgeon or colorectal surgeon), histopathological variables (degree of tumor invasion into the intestinal wall, number of lymph nodes resected, number of compromised lymph nodes, histological tumor differentiation degree, vascular, lymphatic and perineural invasion, surgical margins status [proximal, distal and radial], and TNM stage), and postoperative aspects (length of hospital stay, readmission, clinical complications, and mortality). The results found for each of these variables were always compared between the two periods mentioned above 2018/2019 and 2020/2021.

Analysis of the data was performed using Statistical Package for Social Sciences software v. 13.0 (SPSS Inc., Chicago, Illinois, United States). The Mann-Whitney *U* test was used to analyze continuous variables. Variables such as age and gender were compared with the Student’s *t-*test. Pearson’s chi-square (χ²) test was applied to evaluate numerical data between the groups. The correlation between the different variables in the two periods considered was analyzed using the Spearman’s test. The level of statistical significance was set at a p-value of less than 0.05.

## RESULTS

A total of 112 patients were included in the present study. Of these, 34 (30.3%) patients were allocated to the group undergoing emergency surgical treatment before the COVID-19 pandemic (2018/2019), while 78 (70.7%) underwent surgery during the pandemic (2020/2021). There were 15 (44.1%) males and 19 (55.9%) females in the 2018/2019 group, while the group 2020/2021 comprised 41 (52.6%) males and 37 (47.4%) females. Table 1 shows that no statistical significance was found between the groups in terms of gender (p=0.41, p>0.05). Even though the total cases considerably increased in the COVID-19 pandemic period, no significant difference in mean age was found between the groups: 2018/2019 was 65.2 years (range 53–83), and 2020/2021, was 66.9 years (range 57–81) (p=0.75, p>0.05). Although in the two periods considered there was a predominance of patients who declared themselves white, there were no significant differences (p=0.71, p>0,05) regarding ethnic aspects (Table 1).

**Table 1:** Demographic, perioperative and operative data of the patients.

	Group before pandemic (2018/2019)	Group during pandemic (2020/2021)	p-value
Number of patients (%)	34/112 (30.3)	78/112 (70.7)	
Gender, n (%)			
Male	15/34 (44.1)	41/78 (52.6)	0.41
Female	19/34 (55.9)	37/78 (47.4)	
**Age, mean (min–max)**	65.2 (53–83)	66.9 (57–81)	0.75
Ethnicity, n (%)			
Caucasian	32/34 (98)	74/78 (94.8)	0.71
Black	0/34	3/78 (3.80)	
Oriental	2/34 (2)	1/78 (1.28)	
Clinical presentation, n (%)			
Weight loss	27 (79.4)	69 (88.5)	0.20
Bleeding	11 (32.4)	26 (33.3)	0.91
Abdominal pain	34 (100)	72 (92.3)	0.09
Tenesmus	5 (14.7)	24 (30.8)	0.07
Comorbidity, n (%)			
Diabetes	6 (22.2)	14 (21.2)	0.91
Dyslipidemia	2 (7.4)	3 (4.5)	0.57
Smoking	2 (7,4)	-	
Hypertension	12 (44.4)	36 (54.5)	0.37
Renal failure	1 (3.7)	2 (3.1)	0.86
Reason for surgery, n (%)			
Bleeding	-	1 (1.3)	0.07
Perforation	7 (20.6)	5 (6,4)	
Obstruction	27 (79.4)	72 (92.3)	
Type of procedure, n (%)			
Laparotomy	31/34 (91.2)	65/78 (83.3)	0.51
Laparoscopy	3/34 (8.8)	13/78 (16.7)	
Stoma, n (%)			
Yes	19/34 (55.9)	42/78 (53.8)	0.84
No	15/34 (44.1)	36/78 (46.2)	
Surgeon specialist, n (%)			
General surgeon	8/34 (23.5)	29/78 (37.1)	0.15
Colorectal surgeon	26/34 (76.5)	49/78 (62.8)	

The principal previous symptoms related by the patients before hospital admission were abdominal pain, weight loss, tenesmus, and rectal bleeding in both periods, with no significant difference between them. The results of laboratory tests revealed no significant difference in the serum levels of hemoglobin and hematocrit (p=0.684 and p=0.39, respectively) between the periods, and the serum levels of albumin in the 2018/2019 period were 3.4 g/dL and 3.1 g/dL in 2020/2021, also without a significant difference (p=0.09). The average CPR values were 101.1 mg/dL before the pandemic and 68.8 mg/dL during the pandemic. When comparing these values, no significant difference was identified (p=0.39). Regarding previous comorbidities before surgery, no significant differences were identified when comparing the two periods (Table 1).

When comparing the reason for the emergency surgical indication, it was identified that before the COVID-19 pandemic, 79.4% (27/34) presented intestinal obstruction, and 20.6% (7/34) presented intestinal perforation. In the group of patients hospitalized during 2020/2021, 1.3% (1/78) of patients had surgical indications due to gastrointestinal bleeding, 6.4% (5/78) due to intestinal perforation, and 92.3% (72/78) due to intestinal obstruction (p=0.07, p>0.05).

Prior to the pandemic, 23.5% (8/34) of patients were operated on by general surgeons, while 76.5% (26/34) were operated on by surgeons specialized in colorectal surgery. During the pandemic, 37.1% (29/78) of patients underwent surgical treatment by general surgeons, while 62.8% (49/78) underwent surgical treatment by surgeons specialized in colorectal surgery. There was no significant difference between the two periods (p=0.15, p>0.05).

When considering the access route used to perform the surgical procedure, laparotomy was the most used regardless of the period considered. During 2018/2019, 91% (31/34) of patients underwent laparotomy surgery, while during 2020/2021, the percentage was 83.3% (65/78), with no significant differences between the periods (p=0.51). When analyzing the need to make a stoma during surgery, it was identified that prior to the COVID-19 pandemic, there was a need in 55.9% (19/34) of patients, while in those operated on during the pandemic, a stoma was performed in 53.8% (42/78). When comparing the periods, no significant difference was found (p=0.84, p>0.05). As for the presence of intraoperative complications, it was identified that in the group operated previously to the pandemic, the complication rate was 17.6% (6/34), while in the group of patients operated on during the pandemic, complications occurred in 19.2% (15/78). Comparing the two periods, there was no significant difference (p=0.84, p>0.05).

Table 2 shows the main pathological variables comparing the periods before and during the COVID-19 pandemic. In the period 2018/2019, tumors located in the proximal colon were found in 44.1% (15/34) of patients, and the same percentage was found in the distal colon; in 11.8% (4/34) of patients, the tumor was located in the extraperitoneal rectum. During 2020/2021, 40.2% (30/78), 30.8% (24/78), and 30.8% (24/78) of tumors were located in the proximal colon, distal colon, and extraperitoneal rectum, respectively. According to the localization of the tumor, in the colon or rectal segments, we verified that no differences were identified between the patients operated on in any of the periods (p=0.09, p>0.05).

**Table 2:** Pathological characteristics data.

	Group before pandemic (2018/2019)	Group during pandemic (2020/2021)	p-value
Tumor localization, n (%)			
Proximal colon	15/34 (44.1)	30/78 (40.2)	0.09
Distal colon	15/34 (44.1)	24/78 (30.8)	
Extraperitoneal rectum	4/34 (11.8)	24/78 (30.8)	
TNM staging, n (%)			
I	-	1/78 (1.3)	0.37
II	6/34 (17.6)	15/78 (19.2)	
III	15/34 (44.1)	22/78 (28.2)	
IV	13/34 (38.2)	40/78 (51.3)	
Lymph nodes resected, n mean (min-max)	27 (10–118)	23 (15–49)	0.75
Tumor invasion, n (%)			
T1	-	-	0.67
T2	-	-	
T3	21/34 (61.7)	38/78 (48.7)	
T4	13/34 (38.3)	40/78 (51.3)	
Lymph node involvement, n (%)			
N+ (not resected)	3/34 (8.8)	8/78 (10.2)	0.56
N0	6/34 (17.8)	15/78 (19.3)	
N1	12/34 (35.2)	20/78 (25.7)	
N2	13/34 (38.2)	35/78 (44.8)	
Histological differentiation			
Well	-	4/78 (5.1)	0.28
Moderate	31/34 (91.1)	57/78 (73)	
Poorly	3/34 (8.9)	9/78 (11.5)	
Vascular invasion, n (%)			
Yes	10/34 (29.5)	48/78 (61.5)	0.01*
No	24/34 (70.5)	30/78 (38.5)	
Perineural invasion, n (%)			
Yes	11/34 (23.5)	25/78 (32.0)	0.02*
No	16/34 (47)	15/78 (19.2)	
Lymphatic invasion, n (%)			
Yes	11/34 (32.3)	25/78 (32.0)	0.02*
No	23/34 (67.7)	15/78 (19.2)	
Status of surgical margins, n (%)			
Committed	12/34 (35.2)	3/78 (3.8)	0.19
No-committed	22/34 (64.7)	38/78 (48.7)	

* significant. Mann Whitney *U* test; TNM: tumor-node-metastasis.

In both groups, most patients belonged to stages III and IV of the TNM classification. Before the COVID-19 pandemic, no patients were stage I, 17.6% (6/34) were stage II, 44.1% (15/34) were stage III, and 38.2% (13/34) were stage IV. During the pandemic period, 1.3% (1/78) presented stage I, 19.2% (15/78) stage II, 28.2% (22/78) stage III, and 51.3% (40/78) stage IV. Despite the increase in patients stratified into stage IV disease during the pandemic, there was no significant difference when comparing the two periods (p=0.37, p>0.05) (Table 2).

It was identified that in the group 2018/2019, the mean number of lymph nodes resected was 30.3 per patient, while in the group of patients who underwent surgery on 2020/2021, this number was 26.8. When comparing the mean number of lymph nodes resected in the two periods, no significant difference was found (p=0.65, p>0.05). In regards to lymph nodes resected before the COVID-19 pandemic (2018/2019), it was observed that in patients operated on by general surgeons, the number was on average 19.8 lymph nodes, while in those operated on by colorectal surgeons, this number was 32.5. Despite the higher number of lymph nodes in the surgical specimen in patients operated on by colorectal surgeons, no significant difference was found (p=0.06, p>0.05). In the group of patients who underwent surgery during the pandemic (2020/2021), the mean number of lymph nodes resected by general surgeons was 23.3, while by coloproctologists, 30.6 lymph nodes were resected per patient. When considering the pandemic period, it was found that patients operated on by specialists had a significantly higher number of lymph nodes in the surgical specimen (p=0.03, p<0.05, data not shown). When comparing the total number of lymph nodes compromised in the patients, it was identified that in the period prior to the COVID-19 pandemic, the mean number of lymph nodes compromised was 3.29 per patient, while in the group of patients operated on during the pandemic, this number was on average 6.36 per patient. No significant difference was encountered between the two periods (p=0.65, p>0.05).

In addition, no significant difference was found in the pathological tumor stage (T) between the two groups (p=0.67). Before the COVID-19 pandemic, none of the operated patients had T1 or T2 tumors. However, 61.7% (21/34) had T3 tumors, and 38.3% (13/34) had T4 tumors. During the pandemic period, 48.7% (38/78) of patients had T3 tumors, and 51.3% (40/78) had T4 tumors. In the same way as what happened in the pre-pandemic period, we did not find patients with T1 and T2 tumors (Table 2).

Similarly, no significant differences were found when comparing lymph node status between the two considered periods (p=0.56). In the pre-pandemic period, 17.8% (6/4) did not have lymph node involvement (N0). A total of 35.2% (12/34) of patients were classified as the N1, and 38.2% (13/34) of patients were classified as the N2. During the COVID-19 pandemic, 19.3% (15/78) did not have compromised lymph nodes, 25.7% (20/78) were classified as N1, and 44.8% as N2 (35/78).

It was noticed, as occurs in patients undergoing elective surgery, that in both periods 2018/2019 and 2020/2021, most patients had moderately differentiated tumors. Well-differentiated tumors were not found in the group 2018/2019 but were observed in 5.1% (4/78) of patients during 2020/2021. Moderate tumors were found in 91.1% (31/34) of patients before the pandemic period and in 73% (57/78) during the pandemic. Poorly differentiated tumors were found in 8.9% (3/34) before the pandemic and in 11.5% (9/78) during the pandemic of COVID-19. When comparing the two periods in relation to histological degree, no statistically significant difference was encountered (p=0.28, p>0.05).

Regardless of the presence of vascular invasion, it was identified that in the period prior to the COVID-19 pandemic, 29.5% (10/34) of patients had vascular invasion, while in patients operated on during the pandemic, it was present in 61.5% (48/78) of patients. There was a significant increase in the number of patients with vascular invasion operated on during the pandemic (p=0.01, p<0.05). Likewise, when comparing the presence of lymphatic invasion from tumors, it was identified that in the period 2018/2019, 32.3% (11/34) of patients had lymphatic invasion, while in patients operated on during 2020/2021, the percentage was 62.8% (49/78). There was a significant increase in the number of patients with lymphatic invasion operated on during the pandemic (p=0.02, p<0.05). Similar to what occurred with lymphatic invasion, 32.3% (11/34) of patients had perineural invasion in the period prior to the COVID-19 pandemic and 62.8% (49/78) during the pandemic. When comparing the two periods, it was identified that there was a significant increase in the number of patients with perineural invasion operated on during 2020/2021 (p=0.02, p<0.05). Regarding the presence of compromised surgical margins in both periods, no increase was identified in the number of patients who underwent surgery during 2020/2021, even though these patients presented tumors with worse staging (p=0.19, p>0.05).

Table 3 shows the postoperative variables. In the period prior to the COVID-19 pandemic, patients were hospitalized for an average of 7.8 days, while the group of patients operated on during the pandemic remained hospitalized for 8.5 days. This finding shows no significant difference (p=0.41, p>0.05). As for the presence of clinical complications, it was observed that in the period 2018/2019, 41.7% of patients had at least one clinical complication, while in the group of patients operated on in 2020/2021, clinical complications occurred in 44.9% of patients (p=0.83, p>0.05). In addition, the percentage of patients who needed readmission was 5.9% before the pandemic and 11.5% during the pandemic. However, no significant difference was seen between the periods (p=0.35, p>0.05).

**Table 3:** Postoperative variables.

	Group before pandemic (2018/2019)	Group during pandemic (2020/2021)	p-value
**Length of hospital stay, days**	6	7	0.41
Intraoperative complications, n (%)			
Yes	6/34 (17.6)	15/78 (19.2)	0.83
No	28/34 (82.4)	63/78 (80.8)	
Postoperative complications, n (%)			
Yes	16/34 (47)	35/78 (44.9)	0.83
No	18/34 (53)	43/78 (55.1)	
Readmission, n (%)			
Yes	2/34 (5.9)	9/78 (11.5)	0.35
No	32/34 (94.1)	69/78 (88.5)	
Mortality, n (%)			
Yes	9/34 (26.5)	22/78 (28.2)	0.85
No	25/34 (73.5)	56/78 (71.8)	

When comparing the percentage of patients who died due to surgical or postoperative clinical complications, it was identified 26.5% (9/34) of cases in the period prior to the COVID-19 pandemic and 28.2% (22/78) during the pandemic. There was no significant difference in the number of deaths between the two periods analyzed (p=0.85, p>0.05).

## DISCUSSION

The COVID-19 pandemic required a series of measures from public and private healthcare systems to address the increasing number of infections. These healthcare systems focused mainly on the management of patients infected with the SARS-CoV-2 virus, and as a result, oncological diseases were neglected during the pandemic period^15^.

It has been demonstrated that during this period, surgeons faced with patients presenting greater complications and disease at more advanced stage in consequence of delayed presentation at healthcare facilities. In addition to irreversible complications due to these delays, there were an increase in morbidity and mortality rates, a longer duration of hospital stay, and higher patient costs. In 2021, Brazil published the results of a study based on data provided by the Brazilian Unified Health System (SUS)^27^. The authors showed that between the period before and during the pandemic, there was a substantial reduction in the number of hospitalizations for cancer in Brazil, both clinically and surgically^27^. The same authors also pointed out a difference in the distribution of hospitalizations for cancer between the different states of the country^27^. When comparing the pre-pandemic period and during the COVID-19 pandemic (2019/2020), they found that this reduction negatively impacted the appropriate treatment provided to these patients, especially in the less developed states^27^. Given the delay in the diagnosis and follow-up of cancer patients, a marked impact on the delay of surgical treatment was identified, with increased mortality owing to the progression of neoplasia. It was estimated that for each month of delay in the treatment of CRC patients, there was an increased risk of mortality from 1.06 to 1.08^18,25^.

The need to alter the financial flow for equipment, emergency care centers for severe cases of COVID-19, and the deficiency of beds for the hospitalization of CRC patients, added to social distancing, resulted in the suspension of elective procedures worldwide, including routines for the diagnosis of cancer patients^37^. Similarly, Brazilian oncologists had to modify the flow in the care of cancer patients to minimize the risk of exposure to SARS-CoV-2 virus infection, which also contributed to the progression of CRC^38^. This same strategy was adopted for cancer patients followed up in our reference center.

Alam et al. reviewed the evolution of CRC cases in Lebanon during the COVID-19 pandemic period (2020/2021)^2^. They reported that the group at risk with neoplasm that most requested emergency treatment was patients over 60 years of age, with immunosuppression, cardiovascular comorbidities, diabetes, and chronic respiratory diseases^2^. A retrospective study during the pandemic that included 360 hospitals in the USA analyzed the most Coronavirus-related neoplasms that had the worst clinical outcomes^45^. The group found a significantly higher risk of COVID-19 infection in Afro-descendants than in the Caucasian population. Even though they showed that there was a higher risk group for infection in patients with CRC, they did not analyze the larger portion that needed emergency surgical intervention^45^.

Regarding the main symptoms reported by patients with complications of CRC, emergency treated during the pandemic, it was found that the modification of intestinal habit, hematochezia (bleeding related to evacuation), tenesmus, and, mainly, abdominal pain were the most frequent^39,41^. These data were quite similar to those found in the present study. No differences were identified when comparing the symptoms presented by patients treated in our hospital in the periods before or during the COVID-19 pandemic. The complaints were the same and in a similar proportion. Another important clinical data that negatively influences the prognosis of patients with CRC is the amount of weight loss. This is a multifactorial problem in CRC patients^22^. Cachexia in patients with CRC in more advanced stages is tied not only to isolated weight loss but also to muscle loss, atrophy of adipose tissue, catabolic activity, and systemic inflammation.

Iron deficiency anemia is a common situation in CRC, especially in patients who develop localized neoplasia in the proximal colon^4^. The main laboratory tests that reflect a patient’s anemia are hematocrit and hemoglobin. An Australian study, when analyzing CRC patients who underwent urgent surgical intervention in the presence of anemia, showed that these patients had a higher incidence of postoperative complications, as well as an increase in hospital stay^35^. In the present study, regardless of the period considered (before or during the pandemic), there were no significant differences in serum hemoglobin or hematocrit levels. However, in the two periods, the patients admitted to our hospital had some degree of anemia, which may be related to the high number of patients with advanced stages of the disease (stages III and IV).

Malnutrition in CRC is associated with increased postoperative complications and a worse prognosis^16^. In the short term, hypoalbuminemic patients (serum albumin <3.5 g/dL) present with higher rates of postoperative morbidity and mortality, as well as complications related to wound healing, pulmonary and urinary infections, and dehiscence of anastomoses when compared to patients with normal serum albumin levels^46^. In the present study, although the patients had advanced neoplasms, stratified mainly in stages III and IV of the TNM classification, and which presented an evolution to urgent clinical situations, the serum albumin values, on average, were below the values considered normal.

The C-reactive protein (CRP) serum dosage is widely known as an important nonspecific systemic inflammatory marker. Monitoring serum CRP levels is an important factor related to the development of postoperative complications, mainly related to intra-abdominal infection and fistula^26^. A review of the literature has recently shown that CRP levels are linked to worse evolution, greater presence of complications, and lower survival^26^. In this study, no significant differences were found in CRP values before, on the third day, or on the fifth postoperative day when considering patients operated on before and during the COVID-19 pandemic.

Due to the COVID-19 pandemic, many services specialized in the diagnosis and treatment of CRC suspended endoscopic examination schedules. As a result of this suspension of CRC screening tests, as well as its treatment, there was an increase in the number of patients seeking the emergency room, as a matter of urgency and emergency with tumors in more advanced stages of the disease^28^. With this, there was an increase in the number of emergency hospital visits of patients with obstruction or perforation of CRC worldwide^20,28^. In England, it was estimated that the delay in endoscopic screening and diagnostic investigation was more than six months during the pandemic^29^. Although most specialized services in Brazil experienced a similar phenomenon, in the present study, we found a higher percentage of patients who underwent colonoscopy during the pandemic (2020/2021) than before the pandemic (2018/2019). It is likely that these findings result from the largest number of cases of rectal cancer in patients followed up in our hospital, a reference center for the treatment of this disease, who already had a previous colonoscopy diagnosis since they were already undergoing neoadjuvant chemotherapy treatment and were waiting only for the appointment of the elective surgical procedure.

It is acceptable to assume that with the higher difficulty of medical care and consequent delay in the diagnosis and treatment of CRC patients that occurred during the COVID-19 pandemic, there was a greater number of patients seeking specialized services with tumors in more advanced stages. With the progression of CRC staging, it is likely that variables related to worsening of staging (degree of penetration of tumors into the intestinal wall, presence of lymph nodes compromised by the disease, metastases, tumors with higher rates of vascular, lymphatic and perineural invasion) would also increase in the pandemic period. However, these variables did not always aggravate in patients operated on during the pandemic. A recent study showed that there was no increase in the degree of invasion of the colonic wall by the tumor, but there was greater lymph node involvement, worsening of tumor staging, and a greater presence of liver metastases^44^.

Rottoli et al., in 2022, when analyzing patients who needed surgical intervention for CRC during the COVID-19 pandemic, found an increase in T4 tumors^36^. In the period prior to the pandemic, a large percentage of T4 tumors were already indicated in the clinical-radiological evaluation of patients submitted to urgent surgery for the treatment of CCR complications^5^. The radiological signs of these patients did not always translate into tumor pathological infiltration during the histopathological study, sometimes corresponding only to the peritumoral inflammatory reaction and desmoplasia^5^. The intraoperative impression that a tumor appears to be during surgery is an invasive neoplasm (T4); however, on pathological evaluation, this impression is not confirmed. The greater possibility of finding tumors with increased penetration into the colon and rectum wall, as well as desmoplasia during the pandemic period, is related to the inflammatory process because of the longer delay in diagnosis and, mainly, surgical treatment. The authors, after a general evaluation of both periods (before and during the COVID-19 pandemic), did not identify a percentage increase in more advanced stages or palliative surgeries in patients with CRC^5^. Their study identified discrepancies in the number of cases, which may be associated with reduced screening activities and can potentially affect oncological and survival outcomes^5^. Differently, another group found that stratified tumors in the more advanced stages (stage IV) were significantly higher in the group of patients operated on during the pandemic, mainly with the occurrence of liver metastases^44^. The authors suggested that these findings may be related to the delay in the diagnosis and treatment of patients during the COVID-19 pandemic period^44^. Shinkwin et al. showed that there was an increase of 7.4% in the percentage of cases of emergency surgery due to complications of CRC, with greater invasion of the colorectal wall (T4) in 2020 when compared to the biennium 2018/2019^39^. However, when they analyzed lymph node involvement and the presence of distant metastases, they found no difference when comparing the two periods^39^.

There was no significant difference in the number of lymph nodes resected when comparing the periods before and during the pandemic in the present study. However, despite no statistical significance, a greater number of lymph nodes compromised by neoplasia were found, suggesting that there was a progression of the disease in the pandemic period. Similar results have been described by other authors^44^. Notably, in the period prior to the COVID-19 pandemic, most patients were operated on by both general and specialized colorectal surgeons, which did not impact the radicality of the surgery assessed by the number of resected lymph nodes (minimum of 12 lymph nodes), an important variable related to the prognosis of the disease. Similar to the data found in our study, other authors also showed that there was no worsening of lymph node staging (N) when analyzing the global number of dissected lymph nodes and affected emergency procedures for CRC in the pandemic period^44^. Another interesting aspect that occurred in this study during the COVID-19 pandemic period was that although patients were operated on by both general and colorectal surgeons, the number of resected lymph nodes was significantly higher than that in the pre-pandemic period. It is possible that these results may be related to increased surgical supervision by specialists that occurred during the pandemic.

Vascular and lymphatic invasion refers to the involvement of peritumoral lymphatic and venous vascular vessels and is also considered an important step for the development of lymph node metastases. The incidence of vascular or lymphatic involvement is reported in 4.1% and 63.8% of CRC patients, respectively. In isolation, such as perineural invasion, it was also considered a factor with a worse prognosis^8,31^. The presence of vascular or lymphatic involvement was associated with other adverse factors, such as worse pathological stage, lymph node involvement, distant metastasis, worse degree of differentiation, tumor size, and perineural invasion. When other variables related to worsening prognosis in patients with CRC were analyzed, the present study did not find statistically significant differences between the two periods considered (before and during the COVID-19 pandemic) in the degree of tumor differentiation or in the impairment of surgical margins after tumor resection. However, patients operated on during the pandemic had a higher degree of vascular, lymphatic and perineural invasions than those operated on before the pandemic. The highest rates of vascular, lymphatic and perineural invasions were considered variables with the worse oncological prognosis and were related to delayed diagnosis, more aggressive tumors, and CRC diagnosed in more advanced stages. In the present study, vascular and lymphatic invasions also increased in the pandemic period. It is possible that this fact was also related to the greater number of patients in stages III and IV of the disease in the pandemic period. Perineural invasion corresponds to the invasion of existing nerves in the colon or rectum wall by tumor cells. These cells can grow around or through one or more of the neural layers. The incidence of perineural invasion can vary between 9 and 30%, occurring more frequently in CRC tumors at more advanced stages^10^. When the presence of perineural invasion is considered in isolation, it is a criterion of a worse prognosis. It is possible that the highest rates of perineural invasion found in tumors operated on during the COVID-19 pandemic also reflect the largest number of patients operated on with more advanced tumors in this period.

It is already well defined in the literature that surgical resection margins when positive are associated with a high risk of recurrence and lower rates of disease-free survival and overall survival at five years^10^. Among them, the circumferential resection margin (radial margin) is one of the most important, especially in lesions located in the extraperitoneal rectum, due to the high risk of locoregional recurrence and reduced survival. In the present study, when comparing the presence of proximal, distal and radial surgical margins of resected specimens, comparing the periods prior to the pandemic and during the COVID-19 pandemic, we did not identify significant differences, despite patients presenting tumors in more advanced stages during the pandemic (p=0.19, p=0.44 and p=0.34, respectively). Likewise, regardless of the patients, they were operated on by general or colorectal surgeons, and there was no difference in the number of patients with margins compromised in the histopathological study. These findings suggest that surgeons who operated on these patients had adequate training to perform cancer procedures.

The training of the surgeon may influence the oncological outcome of the emergency-operated patient, especially in cases where tumor resection is required. Considering that CRC is an epithelial tumor and has lymphatic dissemination, it is important to highlight that the prognosis and survival of these patients is related to the need for adequate lymphadenectomy during the surgical procedure. Unfortunately, the surgeon does not always have the necessary qualifications in oncological surgery to perform an ideal lymphadenectomy. It is worth remembering that in the emergency room in most countries, the duty is done by general surgeons, trained for the treatment of routine surgical emergencies, but who have little ability to perform a surgical resection according to the necessary oncological principles. A review study evaluating the experience and training of surgeons for the treatment of CRC patients due to minimally invasive access showed that surgeons with a higher volume of oncological surgical cases had lower percentages of complications and that these outcomes were attributed to specialized training^32^. In a different way, a North American study, which evaluated the distribution between general and colorectal surgeons for every 100,000 inhabitants in several counties, even observing a smaller number of specialists compared to the number of general surgeons, did not identify a difference in mortality due to CRC in these regions^3^. Conversely, another American study that analyzed the specific time of cancer-free survival between surgeons specialized in colorectal surgery and general surgeons showed different results^17^. Patients operated on by specialists who presented CRC in stages II and III had higher survival free of recurrence^17^. A retrospective study analyzing patients operated on in urgent situations between 1996 and 2014, due to intestinal obstruction and perforation, identified a significant difference in the appearance of anastomotic dehiscence, stoma, and mortality in procedures performed by a general surgeon when compared to the specialist^9^. It is worth mentioning that, as an independent factor in the prognosis of the disease, the performance of surgery by a general surgeon appears to be associated with higher rates of recurrence at the distance of CRC^9^. These aspects seem to reflect the impact the adequate training of the surgeon has on cancer therapy and the clinical outcome of patients, making it necessary to create training centers and training in colorectal oncologic surgery in university hospitals.

When comparing the surgical procedures performed by general professionals with those performed by colorectal specialists, it was found that there were no significant differences in most variables related to adequate oncological cancer surgical resection. There was only a greater number of resected lymph nodes in the pandemic period in patients operated on by colorectal surgeons (p=0.33, p>0.05). This fact may be related to the large number of patients operated on by colorectal surgeons during the pandemic period and the large number of compromised lymph nodes due to the presence of tumors in more advanced stages. Another aspect that may explain this evidence lies in the fact that the majority of the general surgeons working in the emergency room at HUSF performed their own surgical training where they received the capacity to perform CRC surgery in urgency and emergency situations.

In surgical patients, the most frequent postoperative clinical complications are infections. Respiratory infection is quite prevalent, especially in emergency surgeries that require an exploitative laparotomy or in a patient who is already predisposed to pulmonary infection, as occurs with smokers. Urinary tract and surgical wound infections often occur due to the precarious nutritional conditions that most CRC patients operated on in urgency present. This study showed a percentage of complications above 40%, both in the period prior to and during the COVID-19 pandemic, with no difference between them. This is probably due to the need for urgent intervention in an immunosuppressed patient with significant malnutrition caused by CRC. The need for hospital readmission is related, in most patients, to the development of clinical or surgical complications. In sarcopenic patients operated on with complications of CRC, a greater percentage of readmissions is expected. Uyan et al., in 2022, found an increase in postoperative complications in those patients referred to surgery during the pandemic, but they did not find an increase in mortality rates^44^. Similarly, in the present study, we did not find significant differences in relation to the rate of postoperative complications or increased mortality rates. These findings can be confirmed by a similar length of hospital stay and readmission rate when comparing the periods before and during the COVID-19 pandemic.

Without a doubt, the COVID-19 pandemic will leave sequels and irreparable losses for all people worldwide. However, the pandemic also left some teachings. Health entities showed that in the face of other natural disasters or new pandemics, all public or private health systems must be sufficiently equipped and prepared with viable proposals to cope with the new calamity. It also taught that the training of professionals working on the front line to contain these disasters must be up-to-date, and the medical professionals must be adequately qualified to respond to and readily solve the patient’s illness.

## CONCLUSIONS

The COVID-19 pandemic had an impact on the diagnosis and treatment of CRC patients followed up at a tertiary reference center in the interior of the state of São Paulo. By comparing patients seen before and during the pandemic, it was found that the COVID-19 pandemic increased the rates of complications related to CRC. The pandemic negatively impacted oncological outcomes in patients with CRC who underwent urgent surgery.
